# Book Reviews

**Published:** 1900-01

**Authors:** 


A TEXT-BOOK OF DISEASES OF THE NOSE AND THROAT. By D. BRADEN KYLE, M. D., Clinical Professor of Laryngology and Rhinology, Jefferson Medical College, etc., etc. Octavo, pp. 646, with 175 illustrations, twenty-three of them in colors. Philadelphia: W. B Saunders. 1899.

This work has both the advantages and disadvantages of a single authorship, but if it has less of the many-sided views of manifold authorship it has the convincing quality of earnest individual opinion. The author brings to the study of his special field a broader pathological knowledge than is shown by many writers upon the same theme. Diseases of the nose and throat are so involved with general diseased conditions, that a grasp of general pathological principles is essential for a reasonably clear understanding of their etiology and principles of treatment. Too often this broad basis for special study is wanting in text-books relating to laryngology. Dr. Kyle frequently illustrates local pathological conditions by reference to well known postmortem observations on more accessible organs. Numerous cuts of original or well selected specimens illustrate the pathological anatomy of the conditions described. Indeed the excellent description of the pathology of diseases of the throat and nose constitute the chief value and originality of the work. The style and language of the author are not always as good and clear as they should be to convey the thoughtful ideas they embody. The topical treatment he advises is usually excellent, but his description of surgical procedures often lacks clearness and sufficient minuteness of detail and at times is of an academic rather than a practical character.

The arrangement of subjects are based on pathological as well as anatomical relations, and the analysing captions of chapters are excellent and enable the student readily to classify his acquired information. Most of the chapters on the various diseases will repay careful reading. The pathology of syphilis is admirably and succinctly given. Special stress is laid on the necessity for long continued treatment after the last active manifestation of the disease. The author is properly conservative in his discussion of "nasal reflexes" about which so many untenable statements have been made. Neoplasms of the respiratory tract are well classified and described. That benign growths may become malignant under irritating treatment is repeatedly mentioned. This may well be borne in mind by numerous rhinologists. Diseases of the septum are interestingly described and various methods of treatment are given with much completeness. No mention is made, however, of the frequency with which nasal obstruction in septal deflection is due largely to the accompanying thickening of the convex side of the septum. In a majority of cases a sufficiently patulous nasal chamber can be secured by the removal of this redundancy, without resort to fracture and resetting of the septum.

An important chapter is devoted to diseases of the accessory sinuses of the nose, illustrated by cuts of well chosen sections of the head, some of them original. The author underestimates the value of frontal transillumination as an aid to diagnosis. It is of decided value as an aid to diagnosis of empyema of the frontal sinus, though the various modifying conditions, alluded to by Dr. Kyle, detract from its exactness. The pathology and symptoms of the various sinus diseases are well described. The description of the required operative procedures is indefinite and unsatisfactory and no mention is made of Luc's operation for antral empyema by which drainage is secured through the nasal wall.

The diseases of the lymphoid structures of the pharynx are well considered and illustrated, though we must take issue with Dr. Kyle's positive statements about the effects of habitual mouth-breathing upon the development of the maxillary bones. The adenoids seem to us to have a concomitant rather than a causal relation to the maxillary deformity. Perhaps the greatest defect in this excellent work is the failure to lay emphasis upon the importance of the hypertrophied pharyngeal tonsil as a cause of deafness and recurring earache in children. It is necessary to impress students of medicine with the great frequency with which adenoid disease is a cause of ear disease. This is true not only of the acute ear diseases of childhood, but of intractable impairment of hearing in later life when the diseased lymphoid tissues may have entirely atrophied. The mischief, preventable by prompt treatment in early life, may easily terminate in incurable deafness in middle life. The danger of chloroform anesthesia in the adenoid operation is not mentioned, though the frequency with which deaths have been reported from this cause within the past five years warrant some attention. Diseases of the larynx receive proper attention, but in that subordinate degree that indicate their usual relation to diseases of the higher portion of the air tract.

The publishers have given the book convenient size, clear print, and free illustration, and its appearance predisposes to that favor which examination of its contents confirms. We can recommend Dr. Kyle's book to all medical men interested in the diseases of which it treats.

A Text-Book of Physiology. By Winfield S. Hall, Ph. D. (Leipzig), M. D. (Leipzig), Professor of Physiology, Northwestern University Medical School, Chicago; Member of the American Physiological Society; Fellow of the American Academy of Medicine. Octavo, 671 pages, illustrated with 343 engravings and six colored plates. Philadelphia and New York: Lea Brothers & Co. 1899.

The aim of Dr. Hall in presenting this new text-book for professional favor is to supply the student with a concise statement of the essential facts of physiology, together with the relevant facts derived from chemistry, physics and morphology. In the introduction, he summarises those principles of chemistry and physics which have a general application to physiology, and to each chapter prefixes an abstract of the facts drawn from all three of the basic sciences which are to be applied in the succeeding text. His plan has been to adapt the work to the needs of medical students, and to students in literary and scientific institutions who are preparing for the study of medicine or of physiology as a specialty.

The work is divided into two parts. Part I. treats of general physiology, including the physiology of the cells and the physiology of contractile and irritable tissues, and Part II. deals with special physiology, which is sub-divided into three divisions—namely, (*a*) nutrition; (*b*) moto-sensory activities; (*c*) reproduction. Under these general divisions is embodied the consideration of circulation respiration, digestion, absorption, metabolism, excretion, physiology of the dermal system, sensation (including the special senses), physiology of the nervous system, physiology of the muscular system and reproduction. The style is brief and concise, but the subjects seem to be as fully covered as the requirements of the student demand. The recent advances in physiology have been duly presented and those pertaining to the anatomy and physiology of the nervous system are of special interest. The work appears to be well suited to the purpose for which it is designed and it is to be heartily commended to the students.

---

PROGRESSIVE MEDICINE. Volume III. A Quarterly Digest of Advances, Discoveries and Improvements in the Medical and Surgical Sciences. Edited by HOBART AMORY HARE, M. D., Professor of Therapeutics and Materia Medica in the Jefferson Medical College of Philadelphia. Octavo, handsomely bound in cloth, 440 pages, eleven illustrations. Philadelphia and New York: Lea Brothers & Co.

The volume under consideration presents carefully prepared and exhaustive papers upon the following subjects: Diseases of the thorax and its viscera, including the heart, lungs and bloodvessels, by William Ewart, M. D., F. R. C. P., physician and joint lecturer on medicine, St. George's Hospital, London. Diseases of the skin, by Henry W. Stelwagon, M. D., clinical professor of diseases of the skin, Jefferson Medical College, Philadelphia. Diseases of the nervous system, by William G. Spiller, M. D., professor of diseases of the nervous system in the Philadelphia Polyclinic. Obstetrics, by Richard C. Norris, M. D., instructor in obstetrics, University of Pennsylvania, Philadelphia.

The aim of the editor and publishers of this series is to offer a well arranged, original, condensed abstract of the practical advances made in each department of medicine during the period covered by the digest. How well they have succeeded can only be determined by a careful examination of the books as they appear. In the present instance we must admit that there is great satisfaction in inspecting the work done by each contributor, and this in spite of a prejudice against works of this sort in general.

"Progressive Medicine" has not only overcome these prejudices but compels our unqualified approval and admiration.

KIRKE'S HANDBOOK OF PHYSIOLOGY. Fifteenth American edition. Revision by WARREN COLEMAN, M. D., Professor of Physiology in the Woman's Medical College, New York, etc., and CHARLES L. DANA, A. M., M. D., Professor of Nervous and Mental Diseases in the New York Post-Graduate Medical School, etc. One volume, 856 pages, 8vo. Profusely illustrated by 516 wood-engravings in black and colors, and by chromo-lithographic plate. New York: William Wood & Co.

The fact that Kirke's Handbook of physiology is in its fifteenth edition is sufficient comment on the value and popularity of the work. It has always been one of the favorite college text-books. It is regretable that a book of such acknowledged value should be marred by certain easily corrected defects. The reviser has failed to change page references and the addition of new matter has so enlarged the book that these references to other portions of the work are valueless. In one or two instances carelessness in incorporating new material quite destroys the sense of a paragraph. Many of the plates are so old that their use obscures rather than elucidates the text. it being impossible to learn anything from a dark and hazy picture, which is little more than a blur.

On the other hand, it is but fair to say that many new plates, clear and valuable, have been added, that the type and paper leave nothing to be desired from a mechanical point of view, and that the revision brings the book up to the present day knowledge of this subject.

---

ANNUAL AND ANALYTICAL CYCLOPEDIA OF PRACTICAL MEDICINE. By CHARLES E. DE M. SAJOUS, M. D., and 100 associate editors. Assisted by corresponding editors, collaborators and correspondents. Illustrated with chromo-lithographs, engravings and maps. Volume IV. Infants, diarrheal diseases of, to mercury. Philadelphia, New York and Chicago: F. A. Davis Co. 1899.

This volume continues the scheme of the series on the same lines that the editor originally mapped out. The article on diarrheal diseases of infants, with which the work opens, is a most timely and well-prepared exposition of the subject.

The contribution on insanity from the pen of the late Dr. George H. Rohé is of absorbing interest, and will be sought by alienists everywhere as an authoritative description of all forms of mental aberration that are grouped under the general term insanity. Rohé's definitions are clear and concise, his symptomatology is succinct and instructive, his treatment scientific and humane, while all the collateral points are developed in a manner that indicate a complete knowledge of the subject, which is imparted to the reader with a marvelous power that cannot be misinterpreted. His section on general paresis reads like a story and is told in fascinating language. This was probably Dr. Rohé's last contribution to medicine, and is in every way worthy of the distinguished author.

The other articles in the volume are deserving of careful study. Especially to be mentioned, however, are the following: Malarial fevers, by James C. Wilson and Thomas C. Ahton; locomotor ataxia, by W. B. Pritchard; intubation, by F. E. Waxham; diseases of the liver, by Alexander McPhedran; meningitis, by Charles H. May, and leprosy, by the editor.

This series when complete will constitute one of the most valuable additions to a physician's library in the way of reference works, that has yet been offered to the profession.

---

Twentieth Century Practice. An International Encyclopedia of Modern Medical Science. By leading authorities of Europe and America. Edited by Thomas L Stedman, M. D., New York City. In twenty volumes. Volume XVIII. Syphilis and Leprosy. New York: William Wood & Co. 1899.

This entire volume is devoted to the consideration of syphilis and leprosy. The contributors are Jonathan Hutchinson, of London; Eduard Lang, of Vienna, and Prince A. Morrow, of New York.

The first 369 pages or a little more than half of the book is allotted to acquired syphilis and is written by Professor Lang. As might be anticipated the pathology of this disease is strongly handled by this author. In the absence of knowledge concerning the germ of syphilis, Lang declines to define the disease, and contents himself with enumerating the effects produced by the contagium, as the only means of characterising the disease. The entire monograph is stamped with masterful knowledge of the subject, and is most fascinatingly written, without verbosity or pedantry. It is, perhaps, all in all, the best contribution to the study of syphilis that has yet been presented to the profession of medicine.

The next section, consisting of twenty-eight pages, takes up the subject of inherited syphilis and is written by Jonathan Hutchinson. It will amply repay all doubters, or those who are without definite opinions as to the transmission of syphilis on the lines of heredity, to carefully study Mr. Hutchinson's exposition of the subject, as set forth in these pages. One of the most important questions in regard to inherited syphilis is the dying out of the taint. How long does it require? How may the fact be recognised? When these questions are answered we shall have made substantial progress in this intricate field.

To the consideration of leprosy, 300 pages of the book are assigned and the article is written by Prince A. Morrow. In it he discourses upon all phases of the disease, throwing much light upon its sources and modes of infection, as well as its treatment. The historical part of the section is most interesting, indeed, it is doubtful if a more complete dissertation has ever been presented upon the whole aspect of the disease in modern times.

The volume is one of great interest from cover to cover.

THE URINE AND THE CLINICAL CHEMISTRY OF THE GASTRIC CONTENTS, THE COMMON POISONS AND MILK. By J. W. HOLLAND, M. D, Professor of Medical Chemistry and Toxicology, Jefferson Medical College of Philadelphia. Sixth edition, revised and enlarged. Duodecimo, forty-one illustrations. Philadelphia: P. Blakiston's Son & Co., 1012 Walnut Street.

This laboratory guide has been in use in medical colleges for many years. It is in convenient form for laboratory use, one page on each leaf is left blank for notes, and it contains all the usual tests employed in the examination of the urine and the gastric contents, besides tests for the detection of the common poisons. It also includes the analysis of milk. The book is a very well-arranged and useful manual.

---

INTERNATIONAL CLINICS. A Quarterly of Clinical Lectures on Medicine, Neurology, Surgery, Gynecology and Obstetrics, Ophthalmology, Laryngology, Pharyngology, Rhinology, Otology and Dermatology, and Specially Prepared Articles on Treatment and Drugs. By professors and lecturers in the leading medical colleges of the United States, Germany, Austria, France, Great Britain and Canada. Edited by JUDSON DALAND, M. D. (Univ. of Penna.), Philadelphia, Instructor in Clinical Medicine and Lecturer on Physical Diagnosis in the University of Pennsylvania; Assistant Physician to the Hospital of the University of Pennsylvania, etc.; Fellow of the College of Physicians of Philadelphia. Volume III. Ninth series. 1899. Octavo, pp. x.—298. Philadelphia: J. B. Lippincott Co. 1899.

The topics dealt with in this number are: drugs and remedial agents; treatment; medicine; neurology; surgery, gynecology and obstetrics; ophthalmology and dermatology. The prominent authors are: At home, Drs. Charles G. Cumston, A. A. Eshner, Edward Jackson, Joseph M. Mathews, and John C. Munro; abroad, Adolph Baginsky, Professor Dieulafoy, Ernst Küster, Alfred Robin, Friedrich Schultze and Francis Warner. There is a preponderance of prominent foreigners among the contributors to this issue.

Professor Mathews, of Louisville, as is his custom, contributes a practical lecture upon rectal diseases, this time having especial reference to the treatment of fistula. This is a disease that formerly was the special prey of the quack, but, thanks to Prof. Mathews and his colleagues in this department of medicine, it is now almost completely wrested from their greedy clutches. There are many other practical lectures in this book and it will prove a valuable aid to the practitioner of medicine who is deprived of post-graduate privileges.

---

American Pocket Medical Dictionary. Edited by W. A. Newman Dorland. A. M., M. D., Assistant Obstetrician to the Hospital of the University of Pennsylvania, etc. Containing the pronunciation and definition of over 26,000 of the terms used in medicine and the kindred sciences, along with over sixty extensive tables. Second edition, revised. Philadelphia: W. B. Saunders. 1899.

This excellent dictionary is only to be seen to be approved. The first edition was quickly exhausted, hence a new one that has afforded an opportunity for revision, of which the authors have taken advantage. It is bound in flexible red leather, possesses a thumb index, is well printed in clear-faced type, and is offered at the low price of $1.25. It is one of the best pocket dictionaries extant.

---

A Manual of Materia Medica, Therapeutics, Medical Pharmacy, Prescription Writing and Medical Latin. For the use of students and practitioners of medicine. By William Schleif, Ph G., M. D., Instructor in Pharmacy in the University of Pennsylvania. In one very handsome 12mo volume of 352 pages. Philadelphia and New York: Lea Brothers & Co.

This is the initial volume of a series of "pocket text-books" issuing by the publishers. It is a condensed exposition of the subjects it deals with and when so regarded it meets approbation. It cannot take the place of the standard text-books on materia medica and allied topics, nor is it intended to do so. It, however, does contain sufficient material to fit the needs of the beginner and to serve as a book of quick reference for the practitioner. The book is well printed upon good paper with clear faced type, and is handsomely bound.

The series is edited by Dr. Bern. B. Gallaudet, demonstrator of anatomy and instructor in surgery at the College of Physicians' and Surgeons', New York.

